# Apolipoprotein CIII predicts cardiovascular events in patients with coronary artery disease: a prospective observational study

**DOI:** 10.1186/s12944-020-01293-9

**Published:** 2020-05-30

**Authors:** Julius L. Katzmann, Christian M. Werner, Tatjana Stojakovic, Winfried März, Hubert Scharnagl, Ulrich Laufs

**Affiliations:** 1 Klinik und Poliklinik für Kardiologie, Universitätsklinikum Leipzig, Liebigstraße 20, 04103 Leipzig, Germany; 2 Klinik für Innere Medizin III, Kardiologie, Angiologie und Internistische Intensivmedizin, Universitätsklinikum des Saarlandes, Homburg, Germany; 3 Klinisches Institut für Medizinische und Chemische Labordiagnostik, LKH Universitätsklinikum Graz, Graz, Austria; 4 Klinisches Institut für Medizinische und Chemische Labordiagnostik, Medizinische Universität Graz, Graz, Austria; 5 Medizinische Klinik V, Medizinische Fakultät Mannheim, Universität Heidelberg, Mannheim, Germany; 6 Synlab Academy, Synlab Holding Deutschland GmbH, P5, 7, Mannheim, Germany

**Keywords:** Apolipoprotein CIII, Chylomicron, Cardiovascular disease, Coronary artery disease, Risk factor, Antisense oligonucleotide, Triglyceride, Ultracentrifugation, Oral fat tolerance test

## Abstract

**Background:**

Apolipoprotein CIII (apoCIII) is associated with triglyceride-rich lipoprotein metabolism and has emerged as independent marker for risk of cardiovascular disease. The objective was to test whether apoCIII is regulated postprandially and whether apoCIII concentrations in native and chylomicron-free serum predict future cardiovascular events in patients with stable coronary artery disease (CAD).

**Methods:**

ApoCIII concentrations were measured in native and chylomicron-free serum in the fasting state and after a standardized oral fat load test in 195 patients with stable CAD. Clinical follow-up was 48 months. Chylomicron-free serum was prepared by ultracentrifugation (18,000 rpm, 3 h). The log-rank test and Cox regression analyses were used to investigate the association of apoCIII with recurrent cardiovascular events.

**Results:**

Of the 195 patients included, 92 had a cardiovascular event, and 103 did not. 97% were treated with a statin. No significant changes in apoCIII concentration were observed after the oral fat load test. The apoCIII concentration was associated with event-free survival independent of conventional risk factors. This association reached statistical significance only for apoCIII concentration measured in chylomicron-free serum (hazard ratio [95% confidence interval] for apoCIII above the mean: postprandial: 1.67 (1.06–2.29), *P* = 0.028, fasting: 2.09 (1.32–3.32), *P* = 0.002), but not for apoCIII concentration measured in native serum (postprandial: 1.47 [0.89–2.43], *P* = 0.133, fasting: 1.56 [0.95–2.58], *P* = 0.081). The effects were independent of other risk factors.

**Conclusions:**

ApoCIII concentrations in chylomicron-free serum are independently associated with event-free survival in patients with CAD both in fasting and postprandial state. This findings support considering apoCIII for risk assessment and attempting to test the hypothesis that lowering apoCIII reduces residual cardiovascular risk.

**Take home message:**

Apolipoprotein CIII concentration measured in chylomicron-free serum predicts recurrent cardiovascular events in patients with stable coronary artery disease.

**Trial registration:**

The trial which included the participants of this study was registered at https://clinicaltrials.gov (NCT00628524) on March 5, 2008.

## Background

Apolipoprotein CIII (apoCIII) is produced in the liver and to a smaller extent in the intestine. It resides on apolipoprotein B (apoB)-containing lipoproteins (LDL, intermediate-density lipoproteins [IDL], very LDL [VLDL], chylomicrons, triglyceride-rich lipoprotein [TRL] remnants) and on HDL, between which it is exchanged rapidly [1]. ApoCIII inhibits lipoprotein lipase and hepatic VLDL uptake, and enhances hepatic VLDL secretion, by this increasing TRL levels [2–7]. Proinflammatory and prothrombotic effects of apoCIII have been described [8–10]. Furthermore, apoCIII modifies the effects of other lipoproteins: HDL particles containing apoCIII have been found to be associated with coronary artery disease (CAD) risk, while HDL particles without apoCIII were protective of CAD [11]; and the risk of CAD due to elevated LDL cholesterol appeared mainly to be due to LDL particles containing apoCIII [12], which may be mediated by the above-described mechanisms.

A causal relationship of apoCIII and cardiovascular disease (CVD) is suggested by two Mendelian randomization analyses, in which loss-of-function mutations in apoCIII resulted in 40% lower triglyceride levels and a 40% reduction in CAD risk [13, 14]. Prospective observational studies have shown an association of apoCIII with incident CAD [15, 16], with this association being independent from triglyceride levels in some studies [17]. In the Ludwigshafen Risk and Cardiovascular health (LURIC) study, a J-shaped association between apoCIII and cardiovascular mortality was found [18]. Furthermore, in a meta-analysis of 12 prospective cohort and case-control studies, an association of apoCIII levels and CVD was reported [19].

Serum triglycerides are regulated postprandially. In the available studies, apoCIII was measured in the fasting or the postprandial state. Whether apoCIII concentration changes due to food intake has not yet been investigated systematically in a sufficient number of patients. Furthermore, it is not known whether the association of apoCIII with CVD reflects the exogenous or endogenous pathways of lipid metabolism, whereas the first is mainly represented by the cholesterol and triglyceride content and associated apolipoproteins of chylomicrons, and the latter by the concentration of cholesterol, triglycerides, and apolipoproteins in chylomicron-free serum [20]. This knowledge might improve risk assessment with apoCIII concentration in patients with established CVD in order to identify those at the highest residual risk most likely to benefit from rigorous risk factor control.

This study aimed to investigate the course of the apoCIII concentration after a standardized oral fat load test and to assess whether apoCIII predicts disease progression in CAD patients, comparing native and chylomicron-free serum.

## Methods

This study encompasses the 195 patients from the prospective *Homburg Cream and Sugar* study [21] included lastly. For the main study, institutional review was provided by the ethics committee of the Saarland (Number 170/07) and all participants provided written informed consent. In brief, between February 2008 und July 2009, consecutive patients with angiographically documented clinically stable CAD were enrolled. In all patients, a standardized oral triglyceride tolerance test (OTTT) with 75 g fat (250 mL cream drink) was performed. In patients without medical treatment for diabetes mellitus, an oral glucose tolerance test (OGTT) was performed to test for the absence of diabetes mellitus. Blood samples were collected before and 3, 4, and 5 h after the OTTT. Patients were followed for 48 months. After 12, 24, and 48 months, standardized telephone interviews were conducted to assess for the occurrence of primary end point events. Hospital records were consulted if patients had been hospitalized. The study end points were adjudicated by a blinded end point committee consisting of at least two experienced cardiologists blinded to the results of metabolic testing [21]. The primary end point was the composite of cardiovascular death, hospitalization for acute coronary syndrome or for unplanned, symptom-induced coronary angiography, and any revascularisation including bypass surgery.

### Laboratory analyses

Routine laboratory analyses were carried out in the core facility of the Universitätsklinikum des Saarlandes, Germany [21]. Lipoprotein separation and analysis was performed from frozen serum samples (stored at − 80 °C) at the Clinical Institute of Medical and Chemical Laboratory Diagnostics, Medical University of Graz, Austria.

Lipoproteins (chylomicrons, VLDL, LDL, and HDL) were separated using ultracentrifugation and precipitation methods. First, the chylomicron fraction was separated by ultracentrifugation (18,000 rpm, 3 h). Lipids and apolipoproteins were measured in total serum and in the infranate after ultracentrifugation (chylomicron-free serum). Second, the chylomicron-free serum was separated in VLDL, LDL, and HDL using a combined ultracentrifugation-precipitation method (beta-quantification) [22, 23]. In brief, VLDL were separated by ultracentrifugation (30,000 rpm, 18 h) at a density of 1.0063 kg/L. After ultracentrifugation, the supernate (containing VLDL) was removed and lipids and apoB were measured in the infranate (containing LDL and HDL). Lipids and apoB in VLDL were calculated as difference between chylomicron-free serum and the LDL/HDL fraction. Then, LDL were precipitated with a phosphotungstic acid/MgCl_2_ reagent in the infranate after removal of chylomicrons and VLDL. Lipids were measured in HDL and lipids in LDL were calculated as difference between HDL and the LDL/HDL fraction.

Total cholesterol (TC), free cholesterol (FC), triacylglycerides (TG), and phospholipids (PL) were measured using enzymatic reagents from Diasys (Holzheim, Germany) and were calibrated using secondary standards from Roche Diagnostics (Mannheim, Germany; TC, TG) and DiaSys (Holzheim, Germany; FC, PL). Esterified cholesterol (CE) was calculated as the difference between TC and FC. Non-esterified fatty acids (NEFA) were analysed using an enzymatic reagent (ACS-ACOD method) from Wako Chemicals (Neuss, Germany). Apolipoproteins and lipoprotein(a) were determined by immunoturbidimetry using reagents from DiaSys (Holzheim, Germany) and standards from Siemens (Marburg, Germany; apoAI, apoB, apoE), Kamiya Biomedical (Seattle, WA, USA; apoAII, apoCII, apoCIII), and DiaSys (lipoprotein[a]). All measurements were performed on an Olympus AU680 automatic analyzer. The coefficients of variation (between day) were < 5% (Supplemental Figure).

### Statistical analyses

Categorical values are expressed as percent. Continuous data are expressed as mean (standard deviation). For comparison of normally distributed data (according to Kolmogorov-Smirnov test), the two-sided *t*-test was used; otherwise, the Wilcoxon test was applied. Baseline characteristics were compared with ANOVA and chi-squared test. Correlation was assessed with the Pearson correlation coefficient. The log-rank test was used to examine differences in event-free survival stratified by tertiles of apoCIII concentration. Multivariable Cox regression analyses for apoCIII concentration above vs. below the mean were performed. Tests of the proportional hazards assumption showed that out of all variables, only age had a relevant interaction. For the other covariates, model fit was not improved by using time-dependent interaction terms. Therefore, the models were adjusted for age as a time-dependent variable (interaction term time*age), gender, LDL cholesterol, HOMA index, fasting triglycerides for fasting samples and 5-h triglyceride area under the curve for postprandial samples, respectively, metabolic syndrome, and smoking status.

The analyses were conducted with SPSS software version 20.0. A two-sided *P* value < 0.05 was considered statistically significant.

## Results

The mean age of the 195 patients was 66.9 years, 87.2% of the patients were men. 92 patients (47.2%) had a cardiovascular event during 48 months of follow-up, and 103 did not. Of the 92 cardiovascular events, the majority were symptom-induced coronary angiography (*n* = 81). *N* = 5 patients had non-fatal myocardial infarction, and *n* = 6 patients died of cardiovascular causes. In consequence, *n* = 42 patients received unplanned percutaneous coronary intervention (PCI) and n = 6 patients were surgically treated with aortocoronary bypass operation.

The medication was similar in both groups, 97.4% were treated with a statin. More patients without event during follow-up were actively smoking (23.3% vs. 10.9% of the patients with event; *P* = 0.017). Alcohol consumption in this group was slightly higher (22.3% drinking alcohol more than three times a week vs. 19.6%). 67% of the patients without event had diabetes mellitus compared to 75% of patients with event. The differences in alcohol consumption and diabetes did not reach statistical significance. Fasting triglycerides were higher in patients with event (168.4 [117.7] vs. 135.6 [67.6] mg/dL; *P* = 0.016), total, LDL, and HDL cholesterol were comparable. The baseline characteristics are shown in Table 1.

**Table 1:** Baseline characteristics

	All	No event	Event	***P*** value
**General**				
N =	195	103	92	–
Age in years	66.9 (10.2)	66.3 (11.0)	67.7 (9.2)	0.358
Male	87.2 (170)	88.3 (91)	85.9 (79)	0.380
Received OGTT	56.4 (110)	60.2 (62)	52.2 (48)	0.163
**Medical History**				
Previous myocardial infarction	43.1 (84)	37.9 (39)	48.9 (45)	0.079
Cardiac bypass surgery	13.8 (27)	9.7 (10)	18.5 (17)	0.059
Previous stroke or TIA	9.2 (18)	9.7 (10)	8.7 (8)	0.503
Peripheral artery disease	7.7 (15)	10.7 (11)	4.3 (4)	0.081
**Medication**				
Platelet inhibitors	96.9 (189)	96.1 (99)	97.8 (90)	0.396
ACE inhibitors/ARBs	96.4 (188)	95.1 (98)	97.8 (90)	0.271
Beta blockers	91.8 (179)	88.3 (91)	95.7 (88)	0.054
Statins	97.4 (190)	98.1 (101)	96.7 (89)	0.447
**Clinical characteristics**				
Smoking (active)	17.4 (34)	23.3 (24)	10.9 (10)	0.017
Alcohol regularly	21.0 (41)	22.3 (23)	19.6 (18)	0.384
Positive family history	34.3 (67)	35.9 (37)	32.6 (30)	0.369
Hypertension	95.9 (187)	92.2 (95)	100.0 (92)	0.005
Systolic blood pressure in mmHg	126.1 (15.1)	124.4 (13.8)	128.0 (16.4)	0.095
Diastolic blood pressure in mmHg	74.6 (8.1)	74.0 (8.1)	75.3 (8.1)	0.246
Resting heart rate in min^−1^	66.6 (8.4)	66.8 (8.2)	66.4 (8.8)	0.771
LV ejection fraction in %	62.3 (12.3)	62.7 (11.9)	61.9 (12.8)	0.664
Body mass index in kg/m^2^	28.8 (3.9)	28.4 (4.1)	29.2 (3.6)	0.194
Waist circumference in cm	103.8 (10.5)	103.0 (10.5)	104.8 (10.4)	0.224
Waist-to-hip ratio	1.00 (0.06)	1.00 (0.06)	1.01 (0.07)	0.477
**Metabolic characterization**				
Normal glucose tolerance	29.2 (57)	33.0 (34)	25.0 (23)	0.142
Impaired glucose tolerance	24.1 (47)	24.3 (25)	23.9 (22)	
Diabetes mellitus	46.7 (91)	42.7 (44)	51.1 (47)	
Metabolic syndrome	59.5 (116)	51.5 (53)	68.5 (63)	0.011
Fasting glucose in mg/dL	129.9 (38.2)	128.4 (37.4)	131.7 (39.2)	0.557
Fasting insulin in μIU/mL	10.7 (9.7)	9.9 (7.8)	11.5 (11.4)	0.238
HOMA index	3.42 (4.08)	3.04 (3.29)	3.83 (4,81)	0.177
HbA1c in %	6.0 (1.1)	5.9 (0.8)	6.2 (1.3)	0.063
Total cholesterol in mg/dL	174.9 (38.4)	173.3 (36.3)	176.6 (40.8)	0.550
HDL cholesterol in mg/dL	44.9 (13.5)	44.9 (11.3)	45.0 (15.7)	0.970
LDL cholesterol in mg/dL	106.4 (34.1)	107.0 (33.3)	105.7 (35.2)	0.802
Non-HDL cholesterol mg/dL	129.9 (138.2)	128.4 (37.4)	131.7 (39.2)	0.557
Fasting triglycerides in mg/dL	151.1 (95.7)	135.6 (67.5)	168.4 (117.7)	0.016
Postprandial 5 h-Tg-AUC in mg/dL	1065 (599)	979 (477)	1161 (701)	0.033
C-reactive protein in mg/dL	4.8 (8.7)	4.6 (7.3)	5.0 (10.1)	0.741

Numerical variables are presented as mean (standard deviation), the other variables are % (n), or as otherwise indicated. *OGTT* oral glucose tolerance test, *TIA* transient ischemic attack, *ACE* angiotensin-converting enzyme, *ARB* angiotensin II receptor blocker, *LV* left ventricular, *HOMA* homeostasis model assessment, *Tg* triglycerides, *AUC* area under the curve

The changes in apolipoproteins after the OTTT in native and chylomicron-free serum are depicted in Table 2. Apolipoprotein concentrations were characterized in four states: in fasting state in native (1) and chylomicron-free (2) serum and in postprandial state in native (3) and chylomicron-free (4) serum. After 5 h, mean apoCIII levels showed a non-significant minor increase when measured in native serum and a slight decrease when measured in chylomicron-free serum, corresponding to an absolute increase of 0.2 mg/dL (3.7%) in native serum and a 0.2 mg/dL decrease (0.6%) in chylomicron-free serum (*P* = 0.122 for native serum, *P* = 0.288 in chylomicron-free serum). Apolipoproteins AI, AII, B and E did not change relevantly after OTTT in native and chylomicron-free serum.

**Table 2:** Apolipoproteins after oral fat tolerance test in native and in chylomicron-free serum

	ApoAI	ApoAII	ApoB	ApoCII	ApoCIII	ApoE
**Native Serum**						
0 h	134.2 (26.4)	37.0 (8.5)	82.6 (24.9)	3.1 (1.4)	10.0 (3.7)	11.5 (3.2)
5 h	132.3 (25.1)	36.9 (8.7)	81.1 (25.2)	3.2 (1.5)	10.2 (3.9)	11.8 (3.4)
Absolute change (mg/dL)	−1.9 (15.0)	−0.2 (3.6)	−1.4 (8.4)	0.0 (0.6)	0.2 (1.5)	0.4 (1.4)
Proportional change (%)	−0.6 (13.5)	−0.1 (10.6)	−1.4 (9.7)	1.8 (24.0)	3.7 (23.1)	3.6 (12.6)
*P* value	0.076	0.402^a^	0.019	0.953^a^	0.122^a^	0.001^a^
**Chylomicron-free serum**						
0 h	122.1 (26.8)	32.3 (8.5)	57.3 (20.7)	2.4 (1.2)	7.5 (4.1)	9.6 (2.9)
5 h	121.4 (26.9)	32.0 (8.3)	55.1 (20.1)	2.2 (1.1)	7.3 (4.2)	9.4 (2.8)
Absolute change (mg/dL)	−0.7 (19.7)	−0.4 (5.0)	−2.3 (9.2)	−0.2 (0.6)	−0.2 (1.9)	−0.2 (1.6)
Proportional change (%)	−0.5 (15.9)	−0.2 (18.0)	−1.8 (26.3)	−0.6 (31.4)	−0.6 (37.4)	−0.0 (17.8)
*P* value	0.622	0.268	0.001	< 0.001^a^	0.288	0.190

Values are in mg/dL and presented as mean (standard deviation) if not stated otherwise. *h* hours. ^a^ Wilcoxon test, otherwise *t* test

The fasting and postprandial apoCIII concentrations (as measured in chylomicron-free serum) were significantly associated with body mass index, fasting glucose, diabetes mellitus, and metabolic syndrome. An inverse association was observed with age. In regard to other lipoproteins, there were strong correlations of the apoCIII concentration with triglycerides and total cholesterol. LDL and HDL cholesterol did not show significant correlations with apoCIII (Tables 3 and 4).

**Table 3:** Correlation of apoCIII concentration in chylomicron-free serum fasting and postprandial with baseline characteristics and other lipid parameters

	Fasting		Postprandial	
	***R***	***P*** value	***R***	***P*** value
**Clinical characteristics**				
Age	−0.18	0.012	−0.15	0.041
Body mass index	0.20	0.005	0.21	0.004
**Clinical chemistry parameters in native serum**				
Fasting glucose	0.39	< 0.001	0.32	< 0.001
HOMA index	0.05	0.520	0.07	0.369
HbA1c	0.12	0.098	0.14	0.064
C-reactive protein	−0.11	0.151	−0.08	0.287
Total cholesterol	0.36	< 0.001	0.294	< 0.001
LDL cholesterol	0.14	0.061	0.08	0.301
HDL cholesterol	−0.08	0.263	− 0.07	0.378
Non-HDL cholesterol	0.39	< 0.001	0.32	< 0.001
Remnant cholesterol	0.68	< 0.001	0.63	< 0.001
Fasting triglycerides	0.68	< 0.001	0.63	< 0.001
**Lipid parameters in lipoprotein subfractions**				
Total cholesterol in CFS	−0.09	0.223	0.01	0.933
Triglycerides in CFS	0.24	0.001	0.31	< 0.001
Chylomicron cholesterol	0.46	< 0.001	0.36	< 0.001
Chylomicron triglycerides	0.67	< 0.001	0.53	< 0.001
VLDL cholesterol	−0.08	0.275	0.16	0.024
VLDL triglycerides	0.18	0.012	0.29	< 0.001
LDL cholesterol	−0.13	0.076	−0.08	0.278
LDL triglycerides	0.08	0.303	0.05	0.469
HDL cholesterol	0.20	0.006	0.21	0.003
HDL triglycerides	0.30	< 0.001	0.28	< 0.001

*R*: Pearson correlation coefficient, *HOMA* homeostasis model assessment, CFS: chylomicron-free serum

**Table 4:** ApoCIII concentration in chylomicron-free serum fasting and postprandial stratified by metabolic syndrome and diabetes-related traits

ApoCIII concentrations in metabolic syndrome and diabetes		Fasting		Postprandial	
		ApoCIII	***P*** value	ApoCIII	***P*** value
Metabolic syndrome	yes	8.2 (4.7)	0.003	7.9 (4.8)	0.026
	no	6.4 (2.7)		6.5 (3.0)	
IFG, IGT, Diabetes	yes	8.0 (4.4)	0.013	7.7 (4.5)	0.038
	no	6.3 (3.1)		6.3 (3.2)	

Values for apoCIII are in mg/dL and presented as mean (standard deviation). *IFG* impaired fasting glucose, *IGT* impaired glucose tolerance

The apoCIII concentration was higher in patients with a cardiovascular event during follow-up. This association did not reach statistical significance for the apoCIII concentration measured in native serum (*P* = 0.122 fasting, *P* = 0.095 postprandial). In contrast, the apoCIII concentration measured in chylomicron-free serum was significantly associated with the primary end point. This association was stronger in the postprandial state (*P* = 0.035 fasting, *P* = 0.008 postprandial). The data are shown in Table 5.

**Table 5:** Primary end point for native and chylomicron-free serum fasting and after fat tolerance test

		Primary end point	n =	ApoCIII in mg/dL (SD)	***P*** value
**Native serum**	fasting	No	103	9.6 (3.5)	0.122
		Yes	92	10.4 (3.8)	
	postprandial	No	103	9.8 (3.9)	0.095
		Yes	92	10.7 (3.9)	
**Chylomicron-free serum**	fasting	No	101	6.9 (3.9)	0.035
		Yes	87	8.2 (4.3)	
	postprandial	No	102	6.6 (3.7)	0.008
		Yes	87	8.2 (4.6)	

**Fig. 1:**
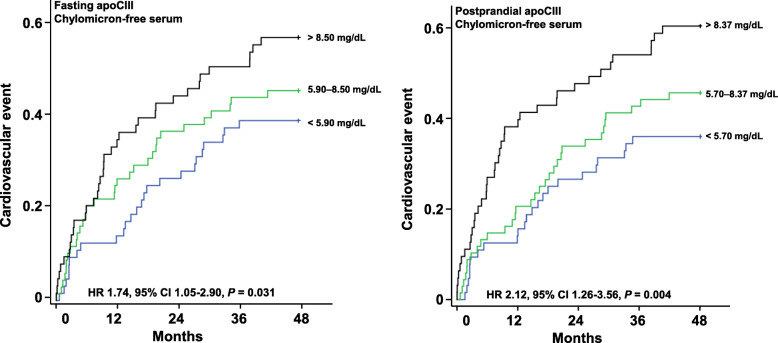
Kaplan-Meier curves of event-free survival for 48 months, stratified by tertiles of apoCIII concentrations in chylomicron-free serum fasting (**a**) and postprandial after standardized fat load test (**b**). HR: hazard ratio, CI: confidence interval

In Fig. 1, the Kaplan-Meier curves for event-free survival stratified by apoCIII tertiles as measured in chylomicron-free serum are depicted in fasting (Fig. 1a) and postprandial state (Fig. 1b). The apoCIII concentration in chylomicron-free serum both in the fasting and the postprandial state was significantly associated with a higher probability of events. This association was stronger in postprandial than in fasting state (hazard ratio [HR] [95% CI] for third vs. first tertile: postprandial: 2.12 [1.26–3.56], *P* = 0.004, fasting: 1.74 [1.05–2.90], *P* = 0.031). If the same analyses were conducted with apoCIII concentration measured in native serum, statistical significance was not reached (postprandial: *P* = 0.060, fasting: *P* = 0.105). In contrast to apoCIII concentration, no other apolipoprotein or other lipid parameters were associated with the primary end point in native or chylomicron-free serum (apoAI, AII, B, CII, E, free fatty acids, lipoprotein[a], free cholesterol, cholesteryl esters, phospholipids; **supplemental Table**).

**Fig. 2:**
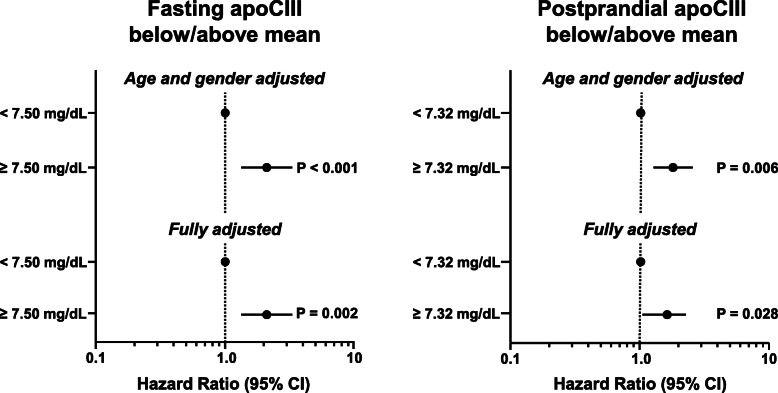
Multivariable Cox regression analyses of the association of fasting and postprandial apoCIII below and above the mean (chylomicron-free serum) and the primary end point (fully adjusted for age*time, gender, LDL cholesterol, HOMA index, fasting triglycerides for fasting samples and 5-h triglyceride area under the curve for postprandial samples, respectively, metabolic syndrome, and smoking status). CI: confidence interval

In multivariable Cox regression analyses, apoCIII concentration above compared to below the mean in chylomicron-free serum was associated with the primary end point when adjusted for age*time and gender (HR [95% CI] postprandial: 1.79 [1.18–2.71], *P* = 0.006, fasting: 2.17 [1.42–3.31], *P* <  0.001). Additionally adjusting for LDL cholesterol, HOMA index, fasting triglycerides for fasting samples and 5-h triglyceride area under the curve for postprandial samples, respectively, metabolic syndrome, and smoking status, apoCIII was associated with the primary end point with a HR (95% CI) of 1.67 (1.06–2.29) postprandial (*P* = 0.028) and 2.09 (1.32–3.32) fasting (*P* = 0.002). In contrast, when measured in native serum, apoCIII concentration above vs. below the mean was not significantly associated with the primary end point (HR [95% CI] postprandial: 1.47 [0.89–2.43], *P* = 0.133, fasting: 1.56 [0.95–2.58], *P* = 0.081). The results for apoCIII measurement in chylomicron-free serum are shown in Fig. 2. The apoCIII concentration in chylomicron-free serum was also associated with the primary end point when apoCIII was used as continuous variable. Per mg/dL increase in apoCIII, in the minimally adjusted model, the HR (95% CI) was 1.06 (1.01–1.11), *P* = 0.019 fasting and 1.02 (1.02–1.11), *P* = 0.007 postprandial, and in the extensively adjusted model, 1.05 (0.98–1.12), *P* = 0.152 fasting and 1.07 (1.00–1.13), *P* = 0.036 postprandial.

## Discussion

This study has two main findings: First, apoCIII concentrations did not change significantly after standardized oral fat intake; and second, a strong association of apoCIII concentration was found in chylomicron-free serum, but not native serum, in the fasting and postprandial state with recurrent cardiovascular events in CAD patients, even after adjustment for conventional risk factors. This findings imply that in CAD patients, apoCIII concentration in chylomicron-free serum may be a superior predictor of disease progression than apoCIII concentration in native serum. Patients with high apoCIII may benefit from rigorous risk factor control.

Changes of the apoCIII concentration after fat intake could have been expected, as previous smaller studies in healthy subjects have shown slight decreases of apoCIII concentration after a duodenal fat infusion (*n* = 10) [24], after oral fat intake (*n* = 16) [25], and after several weeks of diet rich in monounsaturated fatty acids (*n* = 47) [26]. In another investigation of 58 in-patients, no relevant changes in apoCIII concentration during the course of a single day were observed [27]. Similarly, a recent study did not find changes in total apoCIII concentration in *n* = 91 inpatients after a meal [28]. In contrast, in a case-control subgroup of the Leipzig LIFE-Heart study (*n* = 911), apoCIII concentration was 7.3% higher postprandially compared to fasting state [29]. Using a highly standardized metabolic test protocol, no significant changes in apoCIII concentration were observed postprandially, neither in native nor in chylomicron-free serum. A strength of this study compared to the above-mentioned studies is the standardized metabolic test protocol and the high level of metabolic and cardiovascular characterization of the participants.

Despite known effects of glucose as activator of apoCIII expression and of insulin as inhibitor [30, 31], these were not reflected in changes in apoCIII concentration in the patients who had an OGTT (*n* = 110, apoCIII [SD] in native serum: fasting 9.8 [3.6] mg/dL, after test 10.0 [3.6] mg/dL, *P* = 0.071, chylomicron-free serum: fasting 7.4 [4.5] mg/dL, after test 7.4 [4.7] mg/dL, *P* = 0.972). One could speculate that the effects of glucose and insulin counteracted. Alternatively, the standard OGTT test protocol may be too short to detect glucose-induced changes via transcriptional activation or the changes could have been too small to be captured in this study. However, the observed association of diabetes mellitus and apoCIII concentration suggests an interplay between glucose metabolism and apoCIII.

ApoCIII concentrations were associated with cardiovascular events upon follow-up. This association reached statistical significance when apoCIII was measured in chylomicron-free serum, but not in native serum. This indicates differences between the chylomicron-bound proportion of apoCIII and the proportion of apoCIII not attached to chylomicrons. The chylomicron-bound proportion of apoCIII seems to mask the prognostic effect of the proportion of apoCIII bound by lipoproteins other than chylomicrons. After removing chylomicrons, this encompasses especially IDL, VLDL, and HDL [32]. This finding could be explained either by interpreting apoCIII as marker for the associated lipoproteins: chylomicrons cannot penetrate the arterial intima because of their size and are not associated with cardiovascular risk, whereas IDL and VLDL are associated with cardiovascular risk [33, 34]; or by interpreting apoCIII as being atherogenic by itself, whereas its atherogenic properties differ according to the lipoproteins it is bound to. It is likely that the interchange of the apoCIII proportion not bound to chylomicrons between different lipoproteins may also play a role and modulate the associated risk without changing total apoCIII concentration. This observation sets the stage for future studies on apoCIII associated with different lipoproteins [1]. Irrespective of this, and also taking into account that apoCIII was not significantly regulated postprandially, the association of apoCIII and CAD is more likely to be due to the endogenous rather than the exogenous pathways of lipid metabolism.

A similar relationship between apoCIII concentration and cardiovascular risk in CAD patients has been reported previously in two studies. Olivieri et al. reported fasting apoCIII as independent predictor of cardiovascular mortality [35]. A nested case-control analysis from the CARE trial showed that fasting apoCIII concentrations of VLDL and LDL were independent predictors of recurrent cardiovascular events [36]. The HRs for cardiovascular events in the subgroups with high apoCIII in both studies were 2 to 2.5, i.e. remarkably similar to the findings observed in this study. The present study shows that beyond the independent role of triglycerides in predicting cardiovascular events [21], assessment of apoCIII provides additional information to stratify this high-risk population. This may help to identify patients with the highest risk who will, in absolute terms, benefit most from an intervention that reduces residual cardiovascular risk.

Taken together, the current evidence suggests a causal role of apoCIII in CVD. However, it is not clear whether lowering of apoCIII will reduce CVD risk and if apoCIII by itself or the retarded clearance of remnant lipoproteins due to higher apoCIII concentration are causal drivers of CVD [37]. The recent REDUCE-IT study reported that high-dose icosapent ethyl reduces CVD risk in patients with elevated triglycerides [38]. Antisense oligonucleotides that inhibit apoCIII synthesis proved successful in decreasing apoCIII, triglyceride concentrations and other VLDL-associated apolipoproteins [15, 39–41]. These and other emerging therapeutic strategies may allow to test the importance of apoCIII for cardiovascular risk in the future.

### Limitations

A potential limitation of this study is the sample size. Furthermore, statin therapy, which was established in the majority of the patients, may have influenced the observed associations. However, as most CAD patients are treated with statins, this reflects the typical situation in cardiovascular prevention, and underpins that also under statin therapy, apoCIII may be a promising therapeutic target. Strengths include the rigorous metabolic test protocol, the long follow-up, and the prospective design.

## Conclusions

In conclusion, no significant changes in apoCIII concentration after standardized fat load were observed. ApoCIII concentration measured in chylomicron-free, but not native serum, predicted future cardiovascular events in patients with CAD. ApoCIII concentration may be a superior risk marker to predict residual cardiovascular risk when measured in chylomicron-free serum, independently of food intake, however this finding needs to be confirmed in larger studies. Patients with elevated apoCIII may benefit from more rigorous risk factor control. The findings of this study support testing the effects of specific lowering of apoCIII, e.g. with antisense oligonucleotides targeting apoCIII mRNA, on residual cardiovascular risk.

## Supplementary information

**Additional file 1: Figure S1.** Workflow of detailed lipid characterization. * Chylomicron fraction: calculated as difference between total serum and chylomicron-free serum. # LDL fraction: calculated as difference between LDL/HDL fraction (infranate after removal of VLDL) and HDL fraction. Apo: apolipoprotein, TG: triglycerides, PL: phospholipids, FFA: free fatty acids.

**Additional file 2: Table S1.** Kaplan-Meier analyses for apolipoproteins and other lipid parameters.

## Data Availability

The datasets generated and/or analysed during the current study are not publicly available because consent to publish individual participant data was not obtained from the participants of this study. The datasets are available from the corresponding author on reasonable request.

## Abbreviations

apo: apolipoprotein
IDL: Intermediate-density lipoprotein
TRL: Triglyceride-rich lipoprotein
CVD: Cardiovascular disease
CAD: Coronary artery disease
OTTT: Oral triglyceride tolerance test
OGTT: Oral glucose tolerance test
TC: Total cholesterol
FC: Free cholesterol
TG: Triacylglycerides
PL: Phospholipids
EC: Esterified cholesterol
NEFA: Non-esterified fatty acids
