# Supplementary material for: Apolipoprotein CIII predicts cardiovascular events in patients with coronary artery disease: a prospective observational study
Source: Lipids Health Dis. 2020 May 30;19:116. doi: 10.1186/s12944-020-01293-9 (PMC7260843; doi:10.1186/s12944-020-01293-9)
Supplement: Supplementary file 1 — Additional file 1: Figure S1. Workflow of detailed lipid characterization. * Chylomicron fraction: calculated as difference between total serum and chylomicron-free serum. # LDL fraction: calculated as difference between LDL/HDL fraction (infranate after removal of VLDL) and HDL fraction. Apo: apolipoprotein, TG: triglycerides, PL: phospholipids, FFA: free fatty acids. [file 12944_2020_1293_MOESM1_ESM.pptx]

## Slide 1
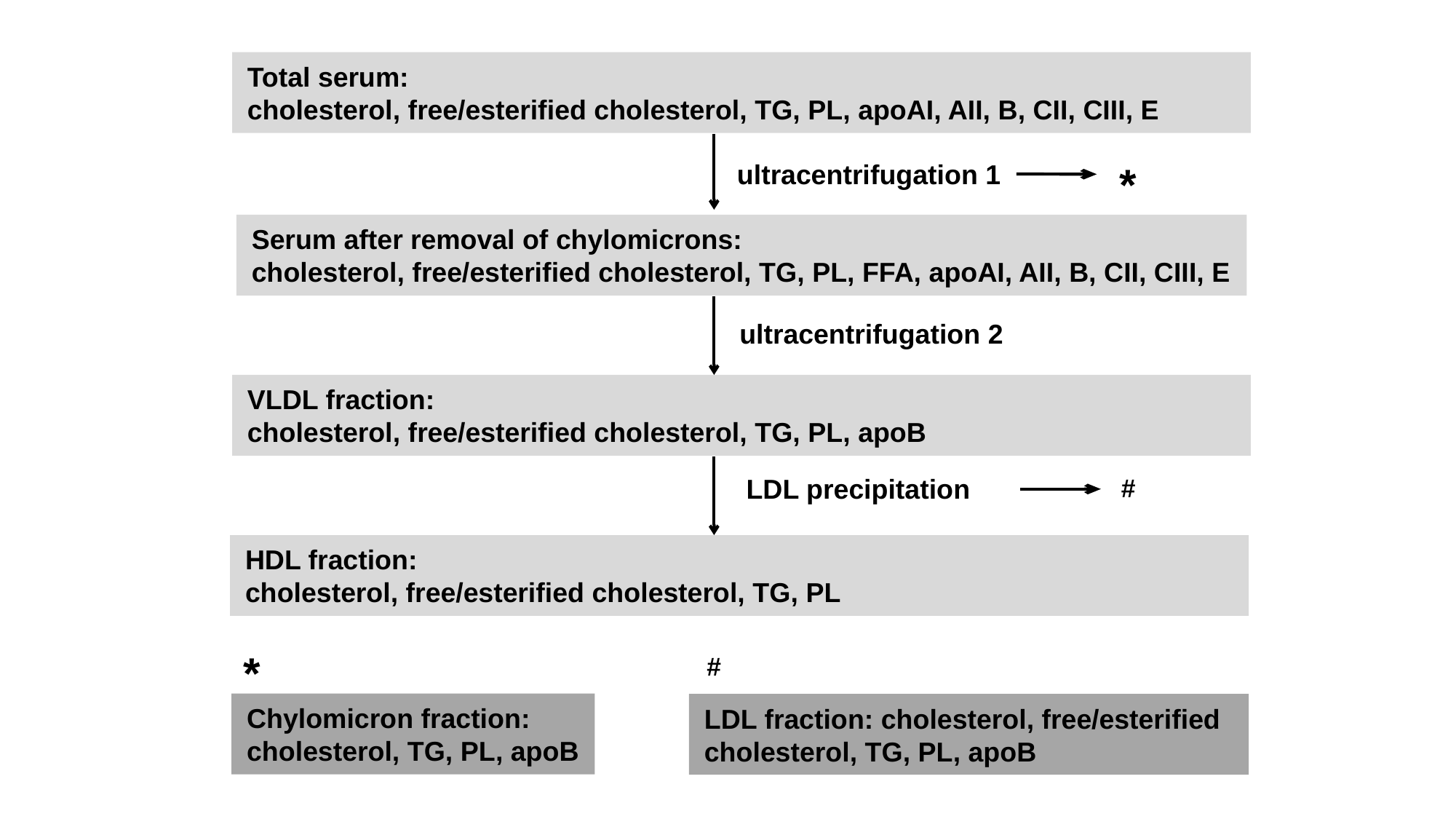

Total serum:
cholesterol, free/esterified cholesterol, TG, PL, apoAI, AII, B, CII, CIII, E
ultracentrifugation 1
*
Serum after removal of chylomicrons:
cholesterol, free/esterified cholesterol, TG, PL, FFA, apoAI, AII, B, CII, CIII, E
ultracentrifugation 2
VLDL fraction:
cholesterol, free/esterified cholesterol, TG, PL, apoB
LDL precipitation
#
HDL fraction:
cholesterol, free/esterified cholesterol, TG, PL
*
#
Chylomicron fraction:
cholesterol, TG, PL, apoB
LDL fraction: cholesterol, free/esterified cholesterol, TG, PL, apoB
