# Supplementary material for: Apolipoprotein CIII predicts cardiovascular events in patients with coronary artery disease: a prospective observational study
Source: Lipids Health Dis. 2020 May 30;19:116. doi: 10.1186/s12944-020-01293-9 (PMC7260843; doi:10.1186/s12944-020-01293-9)
Supplement: Supplementary file 2 — Additional file 2: Table S1. Kaplan-Meier analyses for apolipoproteins and other lipid parameters. [file 12944_2020_1293_MOESM2_ESM.docx]

Supplemental table: Kaplan-Meier analyses for apolipoproteins and other lipid parameters

| Parameter | Native serum | | | | | | Chylomicron-free serum | | | | | |
| --- | --- | --- | --- | --- | --- | --- | --- | --- | --- | --- | --- | --- |
|  | Fasting | | | Postprandial | | | Fasting | | | Postprandial | | |
|  | Mean | Median | Tertiles | Mean | Median | Tertiles | Mean | Median | Tertiles | Mean | Median | Tertiles |
| ApoAI | 0.55 | 0.52 | 0.29 | 0.48 | 0.52 | 0.42 | 0.66 | 0.75 | 0.73 | 0.34 | 0.33 | 0.76 |
| ApoAII | 0.59 | 0.51 | 0.36 | 0.36 | 0.85 | 0.31 | 0.51 | 0.37 | 0.81 | 0.44 | 0.21 | 0.76 |
| ApoB | 0.15 | 0.41 | 0.73 | 0.73 | 0.93 | 0.66 | 0.10 | 0.14 | 0.26 | 0.54 | 0.54 | 0.97 |
| ApoCII | 0.56 | 0.35 | 0.14 | 0.82 | 0.72 | 0.33 | 0.35 | 0.16 | 0.33 | 0.46 | 0.46 | 0.63 |
| ApoE | 0.46 | 0.90 | 0.80 | 0.42 | 0.29 | 0.48 | 0.71 | 0.53 | 0.51 | 0.60 | 0.59 | 0.51 |
| FFA | − | − | − | − | − | − | 0.36 | 0.65 | 0.88 | 0.55 | 0.58 | 0.86 |
| Lp(a) | − | − | − | − | − | − | 0.53 | 0.37 | 0.63 | 0.71 | 0.85 | 0.47 |
| FC | 0.72 | 0.84 | 0.55 | 0.94 | 0.80 | 0.39 | 0.14 | 0.09 | 0.031 | 0.97 | 0.98 | 0.63 |
| CE | 0.33 | 0.78 | 0.87 | 0.47 | 0.88 | 0.69 | 0.23 | 0.17 | 0.16 | 0.66 | 0.68 | 0.86 |
| PL | 0.79 | 0.83 | 0.74 | 0.70 | 0.69 | 0.73 | 0.65 | 0.66 | 0.55 | 0.051 | 0.12 | 0.60 |

*P* values for Kaplan-Meier analyses for apolipoproteins and other lipid parameters above or below the mean, above or below the median, and by tertiles, in native and chylomicron-free serum each fasting and postprandial.

FFA: Free fatty acids, Lp(a): lipoprotein(a), FC: free cholesterol, CE: cholesteryl esters, PL: phospholipids.
